# Radio Frequency Ablation of Enlarged Thyroid Nodules: A Case Report

**DOI:** 10.7759/cureus.73200

**Published:** 2024-11-07

**Authors:** Sabina N Muminiy, Tarek Harhash, Aakash Soni, Aron Yunatanov, Mark Mordukhay, Nathaniel Mavash, Stella Ilyayeva, Benjamin Ilyaev

**Affiliations:** 1 Medicine, St. Francis College, Brooklyn , USA; 2 Medicine, Hofstra University, Hempstead, USA; 3 Medicine, New York Institute of Technology (NYIT) College of Osteopathic Medicine, Old Westbury, USA; 4 Medicine, St. John's University, New York City, USA; 5 Endocrinology and Diabetes, Atlantic Endocrinology and Diabetes, New York City, USA

**Keywords:** large thyroid nodules, radio frequency ablation, thyroid cancer, toxic multinodular goiter (mng), ultrasonography, ultrasound-guided

## Abstract

In the thyroid, abnormal growth can be the result of either benign thyroid nodules (BTNs) or differentiated thyroid cancers (DTCs). If the growth is confirmed to be a DTC, surgical intervention, either a partial or total thyroidectomy, is recommended. Although a majority of BTNs do not require treatment, intervention becomes necessary when nodules cause symptoms, enlargement, or a rare suspicion of malignancy. Clinical presentations of symptoms include psychological and aesthetic ones, such as a large lump on the neck, difficulty swallowing, pressure on the neck, and/or voice changes. The most common form of intervention for minimizing the volume of the BTN is surgery. However, due to several drawbacks to surgery and patient ineligibility, minimally invasive techniques such as radiofrequency ablation (RFA) have been recommended. Radiofrequency ablation serves as a minimally invasive non-surgical procedure that has been recognized to decrease the volume of BTNs.

In the following case, we present a 50-year-old female patient with a growing multinodular goiter (MNG). A thyroid fine needle aspiration (FNA) biopsy and genetic testing confirmed the diagnosis of thyroid disease with possible malignancies. The nodule was increasing in size, causing discomfort, and possible malignancies were indicated by the FNA results. In order to avoid surgical intervention, the patient chose to undergo RFA, effectively decreasing the size of the BTN and dissipating the symptoms. This case highlights the effectiveness of RFA as a non-surgical alternative for treating benign MNG. Additionally, this case study provides insight into the unique treatment plans available for patients who opt out of surgery.

## Introduction

Benign thyroid nodules (BTNs) are discrete lesions on the thyroid gland that are radiologically distinct from the surrounding thyroid parenchyma [1]. Benign thyroid nodules have been becoming more prevalent among thyroid disorders [2]. Their prevalence in the general population has been high, as the percentages depend on the mode of discovery: 2%-6% by palpitation and 19%-35% by ultrasound [3]. The rising incidence of these nodules can be attributed to the extensive use of thyroid ultrasonography, as developmental medical imaging has improved the global detection of thyroid nodules [4]. Thyroid fine needle aspiration cytology (FNAC) is also utilized, and then pathologists utilize the Bethesda system as a post-biopsy (post-FNAC) classification system that categorizes thyroid nodules into six different categories: non-diagnostic, benign, atypia of undetermined significance (AUS), follicular neoplasm, suspicious for malignancy, and malignant [5]. These classifications aid the healthcare team in the risk of malignancy, which further helps with guiding the necessary treatment, intervention, and/or clinical monitoring [6]. 

Several conditions can cause benign thyroid nodules, such as multinodular goiter (MNG), thyroid adenomas, thyroid cysts, Hashimoto’s thyroiditis, Graves disease, colloid nodules, hyperplastic nodules, and thyroiditis [7-8,1]. As this case study focuses on MNG, the development is associated with several additional factors, such as genetic inheritance, gender, elevated thyroid stimulating hormone (TSH), consuming goitrogens, smoking, and stress [9]. Multinodular goiters are structurally and functionally heterogeneous thyroid enlargement, most often caused by iodine deficiency, medication, malnutrition, genetically inherited defects in thyroid hormone synthesis, and growth-stimulating antibodies [10]. The pathogenesis of MNG encompasses multiple processes of focal nodular proliferation, diffuse follicular hyperplasia, and, in rare cases, the eventual development of functional autonomy. The causes of MNG are typically proliferative stimuli that result in insufficient thyroid hormone production, stimulating the anterior pituitary to secrete TSH, a glycoprotein that stimulates the thyroid glands and iodine metabolic pathways by binding to the G protein-coupled receptors (GPCRs) [11]. This binding leads to the activation of the cyclic adenosine monophosphate (cAMP) and phospholipase C pathways, which in turn activate protein kinase A (PKA) and protein kinase C (PKC) downstream, leading to increases in thyroglobulin synthesis, iodine uptake, and production of thyroid hormones (triiodothyronine (T3) and thyroxine (T4)) [11]. Over time, the elevated levels of TSH stimulate the thyroid gland to enlarge and increase its functional mass, resulting in elevated levels of T3 and T4 [11]. 

While the majority of BTNs do not necessarily require direct intervention, it becomes necessary when nodules become symptomatic in patients [12-13]. Various studies have reported that although benign, these nodules can grow about 20% over time. If thyroid nodules undergo sudden rapid growth, whether benign or malignant, it can cause acute respiratory compromise [14]. Multinodular goiter can affect various organ systems, leading to a variety of clinical manifestations. These symptoms can manifest from abnormalities most commonly seen in the endocrine and respiratory systems and rarely in the cardiovascular and nervous systems [10,15]. Common symptoms include difficulty swallowing, tracheal deviation, and cosmetic complaints [10]. Without a proper physical exam, these symptoms can commonly be mistaken or missed entirely. The standard treatments for symptomatic thyroid nodules require surgical intervention to remove the nodules or conservative therapy. However, due to several drawbacks of thyroidectomy, such as risk factors, permanent scarring, and long-term use of levothyroxine postoperatively, patients are hesitant to undergo this treatment [16- 17]. Therefore, there is a need for less invasive options as an alternative to surgery, aiming to reduce the surgical burden on patients. With these clinical manifestations and potential drawbacks of surgery for BTN, radiofrequency ablation (RFA) serves as a safe and effective alternative treatment.

Radiofrequency ablation was first introduced in 2006 and has been known as one of the most effective non-surgical treatments for thyroid nodules. Radiofrequency ablation is a minimally invasive ablation modality for the treatment of thyroid nodules, reporting 60%-90% reduction rates in nodule volume [18]. Multiple studies have reported its efficacy for treating non-functioning BTN and controlling symptoms with decreased complications for two years after RFA [19]. Overall, RFA has been seen as an effective and safe alternative to surgery in high-risk patients and symptomatic patients with BTN [20]. 

There has been a lack of research directly assessing the efficacy of RFA with MNG. The case presented in this paper reflects the positive outcomes and efficacy of the RFA therapy when treating benign MNG. Moreover, this case study provides insight into different treatment plans for patients who opt out of surgical intervention.

## Case presentation

A 50-year-old patient was admitted to the clinic with a previous diagnosis of MNG in 2018. The patient expressed concerns regarding the enlargement of nodules and complained of difficulty swallowing, which was recorded by the physician as feeling an “intermittent tugging sensation on the left side when she swallowed." The patient’s medical history included COVID-19 vaccination, a left foot fracture, fibroid removal in 2008, and the recent RFA of the thyroid in August 2022. Family history included a mother and two out of seven siblings diagnosed with diabetes, but no known family history of thyroid disease. Her social history proved insignificant since the patient had no reported tobacco or medication use and no known allergies. The patient was euthyroid, with no significant changes in weight or appetite and no history of radiation exposure.

The patient presented with a history of MNG, diagnosed in 2018, with the right lobe featuring a hypoechoic nodule at the lower pole and the left lobe with a hyperechoic nodule at the middle pole. Hypoechoic nodules appeared darker on the ultrasound and can sometimes be associated with malignancy, although in this case, the well-defined borders and presence of calcifications suggest a benign process. Despite the normal TSH and the low levels of free T4, the patient was noted as being consistently euthyroid, showing no symptoms of any significant abnormality in thyroid function. These nodules, although benign and recorded as having well-defined borders and calcifications (both being qualities consistent with benign masses), necessitated regular monitoring due to their potential for growth and changes in characteristics, which may lead to indications of malignancies resulting in potential cancers.

Some of the possible diseases are nontoxic MNG, which carries symptoms of an enlarged thyroid gland with multiple nodules, and generally euthyroid, as can be understood from having normal thyroid hormone levels. The patient may also be presenting with thyroid nodules that can manifest as asymptomatic nodules that can be benign or malignant. The diagnosis was concluded through repeated thyroid sonograms and an FNA with additional genetic testing ruling out possible malignancy.

In this case, the repeated sonogram identified nodules in the right and left lobes (Table 1). The nodules appeared as moderately suspicious, with both lobes appearing to have diffusely heterogeneous echotexture consistent with Category IV (Thyroid Imaging Reporting and Data System (TI-RADS) calculator), indicating risk of malignancy. This prompted a FNA biopsy, resulting in a Category III (Bethesda classification system), which indicates AUS in both right and left nodules. A Category III cytology report implicates the malignancy risk and the need to perform ThyroSeq genetic testing (Sonic Healthcare USA ThyroSeq Laboratory, Rye Brook, NY). In this case, the results of the genetic test reported the probability of cancer was low at about 3% (noninvasive follicular thyroid neoplasm with papillary-like nuclear features (NIFTP)).

**Table 1:** The patient's imaging results and operative diagnosis The TSH screening was conducted on August 2021, April 2022, and July 2022, drawing results of 0.9, 0.92, and 0.9 mlU/L, respectively, which had all been within the normal range; however, there had been a concerning increase to 7.2 mlU/L in February 2023 post RFA and then decreased down to 3.46 mlU/L in September 2023, which had been within the normal range. A thyroid sonogram report from September 2021 showed MNG with four nodules in the right lobe measuring 2.6 cm, 1.6 cm, 1.4 cm, and 1.1 cm, respectively. TSH: thyroid-stimulating hormone; FT4: free thyroxine; T-Sono: thyroid sonogram; FNA: fine needle aspiration; MNG: multinodular goiter; RFA: radiofrequency ablation

Date	Imaging modality	Result	TSH (mlU/L) and FT4 (ng/dL) levels
08/2021	T-Sono on 10/2021, FNA done 10/2021 with good results	MNG RL 4 nodules: 2.6 cm, 1.6 cm, 1.4 cm, and 1.1 cm	TSH 0.9
07/2022	No sonogram taken	The patient came to discuss options and wanted RFA instead of surgery	TSH 0.9 FT4 1.0
08/2022	RFA done	RFA on both nodules done	Unknown
03/2023	T-Sono	Right lobe with nodule A 0.8 x 0.6 x 0.5 cm, left lobe with nodule A 1.1 x 0.8 x 1.0 cm	TSH 7.2, FT4 0.8
09/2023	T-Sono	Right lobe with nodule A 0.7 x 0.4 x 0.5 cm, left lobe with nodule A 0.59 x 0.69 x 0.71 cm (classic post-ablative appearance)	TSH 3.46, FT4 0.9

The patient had RFA done in August 2022 with high-frequency alternating electric current oscillating between 200 and 1200 kHz with maximum cell death occurring at temperatures between 50°C and 100°C. On the first follow-up visit, which was in September 2022, the nodules appeared normal in size; however, the patient was still experiencing some discomfort with swallowing. The right nodule measured 19.5 x 13.6 x 18.2 mm, and it was solid with a “classic post-ablative appearance." Additionally, the left midlobe nodule was measured at 23.4 x 14.6 x 22.4 mm. The nodule was described as solid and hypoechoic with irregular borders, heterogeneous, and had microcalcifications. This was noted as a classic post-ablative appearance by the physician. The symptom score had dropped from eight to four; however, the cosmetic score remained at one. The T3, T4, thyroid antibodies, as well as TSH, were in normal ranges.

The patient had another follow-up appointment two months later, in November 2022. She reported that the right side of her thyroid gland became less noticeable; however, the left side presented with a tugging sensation when swallowing. Upon sonogram imaging, the right upper lateral nodule has decreased to 6 x 5 x 4 mm. It was described as solid and hypoechoic with smooth borders, along with being homogenous with no echogenic foci. This is reported as a classic post-ablative appearance; however, there was a note of American Thyroid Association (ATA) intermediate risk, which is indicative of a 10%-20% chance of reoccurrence. The right mid-lower nodule was recorded as being 112 x 8 x 13 mm. It was solid and hypoechoic with irregular borders; also heterogeneous and had microcalcifications. The left midnodule was reported to be 10 x 7 x 9 mm. It was solid and hypoechoic with ill-defined borders. Additionally, the nodule was heterogeneous with microcalcifications. The nodules have undergone significant decreases in size, her symptoms score has gone down from four to two, and her cosmetic score has gone from one to zero. During the thyroid tests, the thyroid antibodies were reported as being abnormal, as well as the free T4.

**Figure 1:**
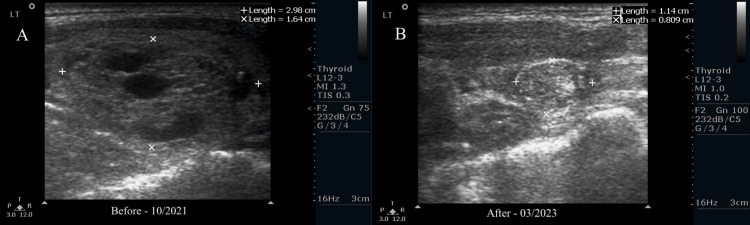
Sonograms from before (A) vs. after (B) RFA Panel A shows the largest left nodule (2.98 cm horizontally, 1.64 cm vertically) before radiofrequency ablation (RFA) was performed. After the procedure, panel B shows the same nodule at less than half its original size (1.14 cm horizontally, 0.809 cm vertically).

The next follow-up was the following year, in September 2023. The right mid-upper lobe nodule was 7 x 4 x 5 mm. It was solid and hypoechoic, having irregular borders and heterogeneous with no echogenic foci. The right mid-medial lobe nodule was measured at 5.9 x 4.9 x 4.5 mm. It was solid, hypoechoic with irregular borders, and heterogeneous with no echogenic foci. The left midlobe nodule had a size of 9.4 x 6.9 x 7.1 mm. It was solid and isoechoic with irregular borders; it was heterogeneous with no echogenic foci. The symptom score dropped to 0, with a cosmetic score going up to one. The endocrinologist recommended annual monitoring of the thyroid. Thyroid testing showed all values returned to normal, with the patient's TSH dropping to 3.46 mL/L (Figure 1).

## Discussion

The patient underwent RFA of the thyroid in August 2022 to address the increasing size of the nodules and associated symptoms. The procedure was successful, resulting in a reduction in nodule size and improvement in the patient's symptoms.

Radiofrequency ablation is a minimally invasive procedure that positively impacts those who are suffering from BTN, which can significantly affect their quality of life. The mechanism of RFA involves thermally induced necrosis of the tissue. The heat is generated from high-frequency electric current oscillating from 200 to 1200 kHz. The procedure is usually performed by surgically inserting a thin electrode into the targeted tissue. High-frequency waves travel down to the electrode tip, agitating tissue around it, which in turn increases the temperature within the nodule [21]. It is essential to note that the temperature within the tissue plays a significant role in the efficacy of the RFA. When the temperature is increased to 42ºC-45ºC, although cells are susceptible to damage by heat and radiation, it is not enough to destroy all the cells; it may even induce more growth following the procedure. By increasing the temperature by several degrees (46ºC-52ºC), the cell undergoes cytotoxicity, where the time necessary to induce cytotoxicity increases with increasing temperature. At temperatures between 60ºC and 100ºC, the cell undergoes immediate, irreversible damage, defined as coagulation necrosis (coagulation of proteins within the cell). However, a temperature greater than 100ºC results in tissue boiling, vaporization, and carbonization of the tissue, decreasing the transmission of the current and thus reducing the efficacy of RFA [22]. 

Current research shows that RFA therapy may result in temporary hypothyroidism and thyrotoxicosis [23]. Similar to our case, other studies show patients with a slight increase in their TSH levels before it decreased to a normal range within the year post RFA [24]. In the case presented, the patient’s TSH levels were elevated above the normal upper limits, which naturally decreased back to the normal range within one year after the procedure.

In addition to the TSH values spiking and then falling post RFA, the levels of thyroid peroxidase antibody (TPO-Ab) had also risen and dropped in many patients undergoing the same procedure. TPO is a crucial enzyme necessary for making thyroid hormones T3 and T4. Increased antibodies to TPO suggest that a patient may have an autoimmune disease called Hashimoto’s disease [25]. These findings suggest that although effective in reducing large nodules, RFA can temporarily cause hypothyroidism such as Hashimoto’s disease. Some symptoms a patient may experience when suffering from Hashimoto’s disease are dry skin, fatigue, hair loss, the presence of goiter, and cold intolerance [26].

## Conclusions

This case study highlights the treatment approach to benign multinodular cysts in the thyroid along with a complication that may arise from therapy. In this case, treatment with RFA was chosen due to it being a safe and minimally invasive procedure. Despite showing a low risk of malignancy, the nodules were increasing in size, and the patient was uneasy with the potential risk of having thyroid cancer.

This procedure had minor complications post ablation, such as a temporary spike in TSH levels as well as thyroid antibodies. Despite this, within a year, the TSH levels and thyroid antibodies went down to normal ranges without medical intervention. The temporary complication that occurred is quite common in the small number of patients that have complications from this procedure. A possible explanation for why this complication occurs is that the RFA causes inflammation leading to cell death and lysis of surrounding thyroid tissue. This in turn causes thyroid peroxidase and other thyroid enzymes to be exposed to the immune system, resulting in a spike in thyroid peroxidase antibodies similar to Hashimoto’s disease. This immune reaction causes more destruction of thyroid hormone-producing cells, which causes the anterior pituitary gland to release more TSH in response to the decreased thyroid hormone production. Eventually, this cascade resolves, leading to the normalization of thyroid hormone and antibody levels.
